# Psychiatric patients’ perspectives of student involvement in their care

**DOI:** 10.1186/s12909-015-0352-z

**Published:** 2015-04-03

**Authors:** Caisa Öster, Susan Bäckström, Ingrid Lantz, Mia Ramklint

**Affiliations:** Department of Neuroscience Psychiatry, Uppsala University, Uppsala, SE-751 85 Sweden

## Abstract

**Background:**

In the education of professionals in psychiatry, one challenge is to provide clinical placements with opportunities for students to interact and have direct contact with patients. The aim of this study was to explore Swedish psychiatric patients’ perspectives on student participation in their care.

**Method:**

In a cross-sectional survey design, 655 adult psychiatric patients at a university hospital completed questionnaires. These questionnaires included statements about student involvement, student gender, attitudes towards student participation as well as two open-ended questions. Data were analyzed quantitatively and qualitatively.

**Results:**

The majority of the patients were comfortable with student participation. There were no differences between patients in wards compared to outpatients but patients who previously had students involved in their care reported higher comfort levels and a more positive attitude. Female patients were less comfortable with male students and very young students. Patients stressed the importance of being informed about the opportunity to refuse student participation. More detailed information given before the consultation as well as the importance of the student showing a professional attitude was conditions that could enable more patients to endorse student participation.

**Conclusion:**

The psychiatric patients’ overall positive attitudes are in line with previous findings from other specialties and countries. The results support both altruistic motives and experience of personal gains by student involvement. More detailed information given beforehand would enable more patients to consider student participation.

## Background

In the education of future health care professionals, involvement in patient-care is a core component. Clinical teaching in outpatient clinics and admission wards is an important part of training. Even though the use of standardized patients and simulations has been introduced, a vital part of learning is through interaction and direct contact with patients [1,2].

Previous research has shown that patients generally accept student involvement in their care [3-8]. A review including studies in general practice concludes that a majority of patients, 83% to 98%, consent to a student’s presence or participation in consultations [9]. The patients’ acceptance of student participation has been related to the type of examination, what kind of illness or problem is being addressed as well as to what degree the student is working alone or with the professional. Patients have reported that the situation is more delicate when students participate in physical exams and are more comfortable when the focus is on gathering information and anamneses [8]. However, patients seem to be less comfortable with students when the consultations include personal or intimate problems [9,10]. More patients are willing to take part in the education process when there is a professional present, especially in the case of more invasive and technical procedures [8,9,11]. Patients would be more positive towards students independently performing the questioning or counseling if they also had the possibility to meet with the doctor alone [6,7].

Patients’ previous experiences of student participation have been related to a more positive attitude towards conceivable student involvement [9,12-14]. Positive first-time experiences of student participation contributed to the patient’s positive attitude towards continuing to allow students to be involved in their care; 95% would let the same student participate, and 90% would let any student participate [15]. Among patients who were negative towards student involvement, a majority had negative past experiences of student participation [5].

Studies report small differences in the patient’s level of comfort with student participation between different clinics and departments [5,16]. However, there is a paucity of studies investigating patients’ experiences and opinions of student involvement in psychiatric care. In one study, by use of a questionnaire, psychiatric in-patients were asked to rate medical students’ effectiveness while training in psychiatry. The results showed high levels of satisfaction with the student-patient relationship, and patients felt that the students made positive contributions to their treatment [17]. In two small questionnaire studies, patients in psychiatric wards were asked about their experiences of participating in interviews in order to teach and train medical students [4,18]. Overall, the patients were positive and would be willing to repeat the experience if asked again [4,18]. Patients with mental disorders from community settings have described specific therapeutic gains in their meetings with students, even though participating was sometimes perceived as distressing [19].

In summary, patients’ perspectives of student involvement in psychiatric care are sparsely investigated. Therefore, the overall aim of this study was to examine Swedish psychiatric patients’ perspectives of student involvement in their care. Specific aims were to explore if psychiatric patients’ attitudes towards students were in accordance with other patients’ attitudes and if there were any differences in patients’ attitudes in relation to patients’ previous experiences with students. Finally, the study aimed to explore patients’ opinions about what could make student participation more comfortable.

## Methods

### Study design

This explorative study followed a cross-sectional survey design, recruiting adult patients at a psychiatric department at a university hospital in Sweden. Medical, nurse and psychology students have their clinical training at the hospital. In the department, patients with substance use disorders, neuropsychiatric disorders, personality disorders, mood and anxiety disorders and psychotic disorders are treated. Participants were recruited from outpatient clinics, inpatient wards and daycare-units. A combination of quantitative and qualitative methods was used for analyzing the responses on the questionnaire.

### Procedure

The investigation was conducted during two weeks in February 2013. A poster with information about the study was posted at each clinic and ward. Administrative assistants or nurses at each clinic or ward distributed questionnaires to all patients registering for appointments or who were inpatients during the study period. The questionnaires were filled in anonymously and returned by the patients in sealed envelopes. There was also an option to return the envelope by post. Information of the study was included in the first section of the questionnaire. Completion and return of the questionnaire was seen as consent to participate. The study was performed in accordance with the principles of research ethics and was approved by the Regional Ethical Review Board in Uppsala 2012/433.

### Assessment

The questionnaire was modified from the study of Passeparuma et al. [16]. From the questionnaire, three statements about student involvement (two concerning student gender) and four statements concerning attitudes were selected. One additional question was changed: instead of asking patients how comfortable they were with a student’s level of education, we asked how comfortable they were with the student’s age. Minor changes were made for better adjustment to the psychiatric context. The English version of the questionnaire was translated to Swedish according to the following steps: (1) translation into Swedish, (2) translation back into English by a native speaker (3) consensus and resolution between the versions. Thereafter, the questionnaire was handed out to six patients in a pilot study. After that, small changes were made for easier and better understanding. The statements assessed comfort levels in terms of student attendance/participation, whether the student performed questioning or counseling together with the professional or if the student independently performed questioning or counseling. Patients used the same scale to value statements of student’s gender and age, questions of how important it is for students to be involved in psychiatric care, if the patient enjoyed the experience with the student, if student participation had positively affected the care, and if the patient would choose a teaching hospital before a hospital without teaching.

There were two open-ended questions tacked on to the questionnaire. One asked what could make student participation in psychiatric care more comfortable for the patient. The second question asked for “other aspects” on student participation, reassuring that aspects not asked about would be paid attention to.

Background data was collected and included the patient’s gender, age, how many times a student had been involved in the patient’s psychiatric care.

The term “student” was used to describe all students from different programs. “Therapist” was used as a title to describe professionals the patient met in psychiatric care such as doctors, nurses, physiotherapists and psychologists. Patients were asked to rate their agreement with each statement using a five-point Likert scale in which “1” denoted strong disagreement, “3” partial agreement, and “5” strong agreement.

### Data analysis

Descriptive statistics were used for the five-point Likert scales. Proportions, mean scores and standard deviations are reported. A higher score indicates a greater comfort level or a more positive attitude. Differences in mean scores for each statement between subgroups were assessed using independent-samples t-tests [20]. The subgroups were based on gender, any previous experience of (or lack of) student participation and age. Participants were divided into three age groups: 18–29 years, 30–65 years and over 65 years. For differences between age groups, repeated-measures of Analysis of Variance (ANOVA) with LD post-hoc analysis were used. The level of significance was set at 0.05. All analyses were performed with the statistical package IBM SPSS 21.0.

Data from the two open-ended questions were managed using Open Code 3.4 software [21] and analyzed using qualitative content analysis, which is a process of identifying, coding and categorizing the primary pattern in the data [22]. Words, sentences and paragraphs expressing the same meaning were identified and coded. Based on commonalities, the codes were sorted into categories [23].

## Results

### Participants

A total of 655 patients returned the questionnaire during the two-week survey. Fourteen questionnaires were sent in by post and arrived within 15 days after the two weeks. The number of patients who were actually invited to participate is not known. However, there were 95 participating in-patients out of 193 registered patients, resulting in a 49% participation rate. The out-patient clinics registered appointments and not individuals. During the two-week study, 5,332 appointments were registered. The number of participating out-patients was 560, approximately 20% of the eligible individual patients. Comparing participating with non-participating inpatients showed that there were no differences in age: mean age 41 ± 16 years versus 37 ± 17 years (t = 1.7, p = 0.09). However, the participating group consisted of more women (61% versus 46%) and fewer men (31% versus 54%) (χ^2^ = 13.4, p ≤ 0.00). Data was missing for sex, age or experience of student participation in up to 14% (n = 94) of the questionnaires. The open-ended questions were answered by 215 (33%) of the responders (67% women, and 33% men).

The participants’ mean age was 36 years (range 18–87). For demographic data, see Table 1. Two thirds of the patients were women, and most of the patients were outpatients. One third of the patients had never had a student involved in their care, while almost one third had experienced more than three occasions with students involved.

**Table 1:** **Descriptive data of 655 participating psychiatric patients**

	No. patients (%)
Total	655
Women	359 (55)
Men	224 (34)
Sex not specified	72 (11)
Age	
Mean (years)	36
Range (years)	18-87
18 – 29 years	241 (37)
30-65 years	301 (46)
>65 years	19 (3)
Age not specified	94 (14)
Outpatients	560 (86)
Inpatients and daycare patients	95 (14)
Prior student involvement during psychiatric care	
Never	206 (32)
In one visit	91 (14)
In two visits	105 (16)
In 3 or > 3 visits	185 (28)
Not specified	68 (10)

### Attitudes towards student participation

#### Comfort levels

**Figure 1:**
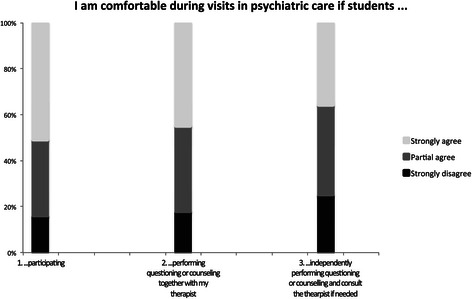
**Patients’ responses to statements related to comfort levels in terms of student involvement.**

The majority of patients strongly or partially affirmed feeling comfortable with student participation (76%), both with the student performing the questions (73%), and with students independently performing questioning or counseling (61%), see Figure 1. For ratings, see Table 2.

**Table 2:** **Patients’ reported comfort levels and attitudes between groups of patients regarding student involvement**

Parameter	Total	Women	Men		Previously met with students	Not met with students	
	Mean (SD)			t value*, *p* value	Mean (SD)		t value* *p* value
*Comfort level with student involvement*							
Attend/participate	3.45 (1.43)	3.31 (1.42)	3.67 (1.38)	−2.99, 0.003	3.65 (1.36)	3.17 (1.45)	4.01, <0.001
Performing questioning or counseling together with my doctor/nurse therapist	3.33 (1.45)	3.25 (1.45)	3.50 (1.41)	−2.08, 0.038	3.57 (1.36)	3.05 (1.49)	4.21, <0.001
Independently performing questioning or counseling and consult my therapist if needed.	2.93 (1.46)	2.79 (1.43)	3.19 (1.46)	−3.27, 0.001	3.17 (1.41)	2.64 (1.46)	4.27, <0.001
*Comfort level with student gender*							
Female students	3.93 (1.40)	3.89 (1.40)	3.99 (1.37)	−0.81, 0.416	4.12 (1.28)	3.71 (1.47)	3.43, 0.001
Male students	3.57 (1.56)	3.38 (1.60)	3.87 (1.45)	−3.66, 0.000	3.71 (1.52)	3.43 (1.58)	2.15, 0.032
*Comfort level with student age*							
A very young student	3.27 (1.51)	3.14 (1.52)	3.47 (1.49)	−2.57, 0.010	3.47 (1.46)	3.03 (1.53)	3.34, 0.001
*Attitudes toward student involvement*							
It is important that students meet patients during their education	4.43 (1.00)	4.43 (1.00)	4.36 (1.07)	0.74, 0.459	4.53 (0.88)	4.34 (1.07)	2.56, 0.024
Enjoyed experiences with students	3.30 (1.36)	3.22 (1.36)	3.43 (1.34)	−1.48, 0.139	3.36 (1.35)	NA	NA
Preference for teaching hospitals	3.34 (1.51)	3.19 (1.48)	3.50 (1.52)	−1.81, 0.071	3.45 (1.46)	3.07 (1.57)	2.16, 0.031
Students increase the quality of care	3.33 (1.31)	3.28 (1.29)	3.38 (1.35)	−0.84, 0.401	3.44 (1.27)	3.22 (1.36)	1.96, 0.051

*Students’ t-test NA = not applicable.

#### General attitudes

Patients’ responses to statements related to attitudes toward student involvement showed mean values between 3.30 and 4.43 (see Table 2). The highest mean value was reported in the statement: “It is important that students meet patients during their education”.

### Group differences

#### Differences between women and men

For comparisons between men and women, see Table 2. Male patients reported higher comfort levels than female patients in terms of student involvement. Regarding female students, there were no differences between male and female patients, but female patients were less comfortable with male students than male patients were. Female patients were also less comfortable with very young students. There were no differences between female and male patients concerning the importance of student involvement in care for educational purposes or the opinion that student involvement had increased the quality of care.

#### Differences in relation to experiences of student participation

Patients who previously had students involved in their care reported higher comfort levels and a more positive attitude in all statements compared to patients who had not had students involved in their care (see Table 2).

#### Differences between age groups

Results of the ANOVA (not shown) with the three groups (18–29 years, 30–65 years and over 65 years) and respective items showed no differences between age groups concerning perspectives on student participation. However, there were group differences according to previous experience of student involvement. This was more common in the group of patients over 65 years old (M = 1.75, SD = 1.23) than in patients under the age of 30 (M = 1.30, SD = 1.24; F (2) = 3.35, p = 0.036).

#### Differences in relation to out- or inpatients

There were no differences in response to statements between outpatients and inpatients. However, previous experience of student involvement was more common in inpatient/daycare (M = 1.76, SD = 1.30) than in outpatient care (M = 1.36, SD = 1.23); t (569) = 2.75, p = 0.006.

### Opinions about facilitators for student participation

The answers (n = 215) on the two open-ended questions asking what could make student participation in psychiatric care more comfortable for the patient as well as the question regarding “other aspects” on student participation were analyzed together. The result of the analyses produced three categories: Conditions making student involvement more comfortable, Inconveniences with student involvement, and Benefits of student involvement.

#### Conditions making student involvement more comfortable

The subject that raised the most comments was related to information. The importance of having information about student participation before the outpatient visits, or the meeting at the ward, was highlighted. “To be informed about if the student is a man or a women and about the student’s age”.

It was also important to feel able to say no as well as to be informed of the possibility to decide for the student to leave the room during the visit. The patients noted that they wanted more information concerning the student’s education (which program and what semester) and more details of how the student was going to be involved. “To be informed of why they are there (the students) and what their role is”.

Student performance was one subject. Some patients thought that the student should be quiet and just attend, while other patients wanted the student to participate, ask questions and take part in the discussions. “It is important that they just don’t sit besides and look at you”.

It was seen as important that the student presented her/himself. There were comments regarding the relationship between how comfortable one is with student participation and what subjects arose in the counseling. There could be subjects perceived as too delicate and intimate for a patient to allow a stranger’s involvement. “It is never easy when there is so much feelings…”. There were also comments about having the possibility to restrict the number of people in the room.

The student’s character was shown to influence how convenient student involvement was perceived. Patients felt more comfortable when the students were engaged, well prepared, without preconceptions, cheerful, positive and with a sound view of human beings.

“The student has to be involved in my care and not studying the interaction between the counselor and me”. Some patients noted that they wanted to know the student’s age and sex beforehand, and some patients did not want new beginners or students that were too young, even though one comment from a young patient stated:

“I would rather have a young student with me who will understand me”.

#### Inconvenience with student involvement

The most noted comment in this category was the fear that confidentiality would be mishandled. Patients did not want to be recognized, as sometimes a person is well known in society. Most of all, if a patient is a student, they run the risk of meeting with fellow students. “Being a patient and a student in this town you are in the same company as the student you meet.”

Some patients proposed that this would be easier if they could have the name of the student beforehand and then decide how to proceed. Some patients declared that it was uncomfortable to meet with an unknown person, and it was annoying to “have to repeat my story over and over again”. There were also some comments stating, “I don’t want students to be involved”.

#### Benefits of student involvement

Student participation sometimes implicated that professionals behaved more professional, and the meetings were more constructive with more time reserved. Many patients wrote that student involvement was fine as it was, and some comments stated that it was a joy to participate in the education of new professionals. The more real patients the students meet with, the better”. Many comments stated that it was important for students to be involved in health care because they have to be taught “empathy and not only theory”.

## Discussion

The majority of the participating adult Swedish psychiatric patients were comfortable with student participation. Patients who previously had students involved in their care reported higher comfort levels and a more positive attitude. Female patients were less comfortable with male students and very young students. Psychiatric patients perceived student participation in similar ways as reported by patients from other specialties and countries. In the open-ended questions, patients stressed the importance of being informed about the students’ participation before the consultation, as well as the importance of the student showing a professional attitude.

Through use of the same questionnaire that Passaperuma et al. used in their Canadian study [16], the patients’ positive attitudes towards student participation could not only be replicated but also comparably quantified. On the five-graded Likert scale, ratings differed very little between the two studies. However, there was a tendency for the psychiatric patients to rate a little lower. The greatest difference was 0.7 points on the item about enjoying the experience with students. However, many of these psychiatric patients suffered from depression or psychotic disorders, which made it difficult for them to feel joy. The state effect as an explanation of the differences is supported by the ratings of the item concerning the importance of using real patients in clinical teaching. Here, the difference was only 0.12. Additionally, the validity of the exact ratings, i.e., the meaning of the difference between this and the Passaperuma study, is not known. It might lack importance. That there should be no difference between psychiatric patients and others is supported by a previous Swedish study [5]. On the other hand, patients in general practice have reported less willingness to let students participate when the consultation is about intimate or delicate problems [9,10]. In other specialties, where patient integrity is more easily violated, such as obstetrics/gynecology, patients have repeatedly reported a more restrictive attitude towards student participation [8,24]. Mental disorders are still connected with social stigma [25], which might explain why the psychiatric patients rated their comfort levels and attitudes somewhat lower.

Patients in wards compared to outpatients are expected to be more severely ill and, therefore, might experience student participation as more threatening. The data do not support this fear. There are several possible explanations for this. Inpatient-care enables continuity in patient-student relations, which has previously been shown to increase patient satisfaction in psychiatric care [4,17]. Furthermore, inpatients reported more experience of contact with students, which have previously been known to positively influence patients’ attitudes towards students [5,9,26].

Female patients were more hesitant towards male students. If this hesitation is related to female psychiatric patients’ higher experience of sexual abuse [27], social structures between the sexes [28] or other causes is not known. Previous research has not focused on gender differences in patients’ attitudes towards students, but Passaperuma [16] found that patients in all specialties, except urology, reported greater comfort with female students than with male students. In other studies, female patients in obstetrics/gynecology departments reported higher acceptance of female students compared to male students [8,11]. In general practice, studies show that female patients preferred a student of their own sex more often than men did [9,10]. The findings have implications for clinical teachers when introducing students to patients. It would be interesting to further examine gender differences of student participation with patients from psychiatric care as well as other specialties.

Inconvenience with student participation was mostly described as fear of insufficient confidentiality. Throughout the patients’ comments, having information about student participation was consistently articulated as important for being comfortable with student involvement. This may be an expression of the patients’ need for autonomy in their role as patients.

To have detailed information of the student beforehand increased the likelihood of the patients to consider student involvement, despite concerns of confidentiality. It was important to be informed of not only the student’s name and year of education, but also the student’s role and to what extent the student was supposed to be involved. These requests are previously expressed by patients in other studies [9,13,29]. Informing patients about student participation is often neglected. Reluctance to seek informed consent may come from a view that if patients are given a choice, they may refuse. In the study by Lynoe et al., 41% of the patients estimated that they once, or on several occasions, had students involved in their care without having been informed [5]. Providing information and asking for consent are often the tasks of the clinical teachers, regardless of student category, and their cooperation with patients is a core component in the clinical learning environment [7]. For the clinical teacher, there can be a balancing act between the individual patient’s right to decline student participation in their care and the preceptor’s commitment to high quality clinical education. Patients’ comfort level decreased when the student was supposed to perform more independent tasks without the clinical teacher participating. This is in accordance with Passaperuma’s findings [16]. Some patients commented that they did not want students involved in their care at all. Tang & Sky found that clinical teachers who rated themselves as more comfortable with informing patients that a medical student would be performing an intimate exam were less likely to encounter patient refusal in the context of a gynecological/urological exam [30]. Other studies from general practice have shown that with written information about student participation in advance, patients felt free to decline compared to being asked at the time of consultation, and it did not negatively affect the inclination to allow student participation [31].

Positive experiences with student participation such as “the professionals behaving more professional” and “it is a joy to participate in education” were highlighted. Patients in hospitals have previously perceived students as having extra time and attention and felt that they were learning more about their conditions [9,11,16,32]. In teaching hospitals, almost half of the patients reported that student involvement increased the quality of health care [8]. In a general hospital, quality of care improved with the presence of medical students [33]. In addition, involvement of inter-professional student teams contributed to patients’ experiences of better involvement in the decision-making process, more satisfaction with information and better preparedness before discharge, compared to ordinary care [34].

The results also support altruistic motives for being positive towards student participation. Patients commented that it was important for students to be involved in health care, and that they have to be taught “empathy and not only theory”. Patients in psychiatric care have, in previous research, perceived the importance to help students learn with real patients [18] and felt they had something important to offer as well as a sense of giving back [19]. Even unprepared patients in general care think of themselves as contributors to and facilitators of learning, particularly in professional skills and attitudes [10]. This learning situation is based on patients’ active participation and can be threatened if students do not see the patients as individuals and subjects [35]. A challenge for clinical teachers is to create an educational atmosphere where students learn *with* and not *about* patients.

The major strengths of the study are the large sample size, wide age range and participants from different psychiatric care units in a university hospital during two weeks of full student activity. Another strength is the use of open-ended questions, where the extent of answers may be interpreted as patients’ willingness to contribute to better learning situations, both for patients and students. Patients’ voices about student involvement are not extensively heard, even less so from psychiatric patients. Furthermore, the inclusion of patients with ad without previous experience of student participation (one third of the patients had no previous experience) most likely contributed to a broader perspective in conditions, making student involvement more comfortable.

Some limitations have to be addressed. First, the questionnaires were filled in anonymously; this did not allow a more detailed comparison with non-responders. The questionnaire has only been used once before, and there is no formal validation study published. Another limitation is the distribution of questionnaires during the two-week investigation. It was handled by administrative assistants or nurses at each clinic or ward. Based on their workload, this could have affected the response rate. This challenged the representativity of the participants.

The questionnaires were answered and returned at the time of the appointments or during the inpatient period, which could have influenced the answers. It can be difficult to honestly rate and comment on situations on which you are dependent. Therefore, anonymous questionnaires and sealed envelopes were used. On the other hand, receiving a questionnaire several months after the experience could have an impact on memories.

Answers could also have been affected by social desirability, i.e., being negative towards student education is not a socially desirable attitude.

More detailed information about barriers against student participation could have been obtained by analyzing data from the participants who were negative towards student involvement, even though they were few.

## Conclusions

The majority of psychiatric patients reported a positive attitude towards student involvement in their care. Sufficient information before the student participation and the opportunity to refuse were conditions that made student involvement more comfortable. More detailed information given beforehand could enable more patients to endorse student participation.

## References

[1] Diemers AD, Dolmans DH, Verwijnen MG, Heineman E, Scherpbier AJ (2008). Students’ opinions about the effects of preclinical patient contacts on their learning. Adv Health Sci Educ Theory Pract.

[2] Chapman L, James J, McMahon-Parkes K (2011). Involving patients in assessmentof students. Nurs Times.

[3] King D, Benbow SJ, Elizabeth J, Lye M (1992). Attitudes of elderly patients to medical students. Med Educ.

[4] Santulli RB (1993). Reactions of psychiatric inpatients to medical student interviews. Acad Med.

[5] Lynoe N, Sandlund M, Westberg K, Duchek M (1998). Informed consent in clinical training–patient experiences and motives for participating. Med Educ.

[6] Hajioff D, Birchall M (1999). Medical students in ENT outpatient clinics: appointment times, patient satisfaction and student satisfaction. Med Educ.

[7] Townsend B, Marks JG, Mauger DT, Miller JJ (2003). Patients’ attitudes toward medical student participation in a dermatology clinic. J Am Acad Dermatol.

[8] Marwan Y, Al-Saddique M, Hassan A, Karim J, Al-Saleh M (2012). Are medical students accepted by patients in teaching hospitals?. Med Educ Online.

[9] Mol SS, Peelen JH, Kuyvenhoven MM (2011). Patients’ views on student participation in general practice consultations: a comprehensive review. Med Teach.

[10] Haffling AC, Hakansson A (2008). Patients consulting with students in general practice: survey of patients’ satisfaction and their role in teaching. Med Teach.

[11] Sayed-Hassan RM, Bashour HN, Koudsi AY (2012). Patient attitudes towards medical students at Damascus University teaching Hospitals. BMC Med Educ.

[12] Cooke F, Galasko G, Ramrakha V, Richards D, Rose A, Watkins J (1996). Medical students in general practice: how do patients feel?. Br J Gen Pract.

[13] Hartz MB, Beal JR (2000). Patients’ attitudes and comfort levels regarding medical students’ involvement in obstetrics-gynecology outpatient clinics. Acad Med.

[14] Mavis B, Vasilenko P, Schnuth R, Marshall J, Jeffs MC (2006). Medical students’ involvement in outpatient clinical encounters: a survey of patients and their obstetricians-gynecologists. Acad Med.

[15] York NL, DaRosa DA, Markwell SJ, Niehaus AH, Folse R (1995). Patients’ attitudes toward the involvement of medical students in their care. Am J Surg.

[16] Passaperuma K, Higgins J, Power S, Taylor T (2008). Do patients’ comfort levels and attitudes regarding medical student involvement vary across specialties?. Med Teach.

[17] Black AE, Church M (1998). Assessing medical student effectiveness from the psychiatric patient’s perspective: the Medical Student Interviewing Performance Questionnaire. Med Educ.

[18] Doshi M, Acharya S, Wall D (2006). Mentally ill inpatients’ experiences and opinions on seeing medical students: a questionnaire study. Med Teach.

[19] Walters K, Buszewicz M, Russell J, Humphrey C (2003). Teaching as therapy: cross sectional and qualitative evaluation of patients’ experiences of undergraduate psychiatry teaching in the community. BMJ.

[20] Norman G (2010). Likert scales, levels of measurement and the “laws” of statistics. Adv Health Sci Educ Theory Pract.

[21] Health IaSaSDaDoEaG (2009). OpenCode, 3.4.

[22] Krippendorf K (2013). Content analysis: an introduction to its methodology.

[23] Graneheim UH, Lundman B (2004). Qualitative content analysis in nursing research: concepts, procedures and measures to achieve trustworthiness. Nurse Educ Today.

[24] Jiang X, Altomare C, Egan JF, Tocco DB, Schnatz PF (2012). The ObGyn clerkship: are students denied the opportunity to provide patient care and what is the role of gender?. Conn Med.

[25] Hogberg T, Magnusson A, Lutzen K, Ewalds-Kvist B (2012). Swedish attitudes towards persons with mental illness. Nord J Psychiatry.

[26] Isaacson JH, Neides D, Mayer M, Nottingham K (2014). Patient perceptions of having 1st- and 2nd-year medical students involved in their care. Teach Learn Med.

[27] Oram S, Trevillion K, Feder G, Howard LM (2013). Prevalence of experiences of domestic violence among psychiatric patients: systematic review. Br J Psychiatry.

[28] Goffman E (1977). The arragemnet between the sexes. Theory Soc.

[29] Tang TS, Skye EP, Steiger JA (2009). Increasing patient acceptance of medical student participation: using interactive theatre for faculty development. Teach Learn Med.

[30] Tang TS, Skye EP (2009). When patients decline medical student participation: the preceptors’ perspective. Adv Health Sci Educ Theory Pract.

[31] Westberg K, Lynoe N, Lalos A, Lofgren M, Sandlund M (2001). Getting informed consent from patients to take part in the clinical training of students: randomised trial of two strategies. BMJ.

[32] Scheffer C, Edelhauser F, Tauschel D, Riechmann M, Tekian A (2010). Can final year medical students significantly contribute to patient care? A pilot study about the perception of patients and clinical staff. Med Teach.

[33] Esguerra R, Toro J, Ospina JM, Porras A, Diaz C, Reyes S (2014). The transition to a teaching hospital: Patient satisfaction before and after the introduction of medical students. Med Teach.

[34] Hallin K, Henriksson P, Dalen N, Kiessling A (2011). Effects of interprofessional education on patient perceived quality of care. Med Teach.

[35] Manninen K, Henriksson EW, Scheja M, Silen C (2014). Patients’ approaches to students’ learning at a clinical education ward-an ethnographic study. BMC Med Educ.

